# Bilateral pleural effusion as an initial manifestation of multiple myeloma: A case report and literature review

**DOI:** 10.3892/etm.2015.2184

**Published:** 2015-01-15

**Authors:** AI-GUI JIANG, YU-TIAN YANG, XIAO-YAN GAO, HUI-YU LU

**Affiliations:** Department of Respiratory Medicine, Taizhou People’s Hospital, Taizhou, Jiangsu 225300, P.R. China

**Keywords:** multiple myeloma, pleural effusion

## Abstract

Multiple myeloma (MM) is a rare type of malignant hematological neoplasm. Although primarily involving the bone marrow, MM has a significant risk of metastasizing to other organs and may present with various clinical symptoms. However, the involvement of the respiratory system in the course of MM is extremely uncommon, particularly presenting with bilateral pleural effusion as the sole initial manifestation, which may result in a delayed diagnosis of MM. The present study describes the extremely rare case of a patient with MM presenting with myelomatous pleural effusion (MPE). The 78-year-old patient was admitted to the Department of Respiratory Medicine, Taizhou People’s Hospital (Taizhou, China) in March 2014, complaining of persistent dyspnea. Following admission, chest computed tomography scans revealed bilateral pleural effusion and a small amount of pericardial effusion, but no evident mass lesion. Thoracentesis was performed and the resulting pleural effusion was exudative and slightly bloody. In the following cytological examination, myeloma cells were identified in the pleural effusion. The patient was diagnosed definitively with MM following a histopathological study of the bone marrow aspiration. Therefore, the observations of the present case report may promote the consideration of MM in the differential diagnosis of patients with unexplained and refractory pleural effusion. The present study also reviewed the literature with regard to the association between MM and pleural effusion.

## Introduction

Pleural effusion may be caused by a variety of diseases, including malignant tumors, pneumonia, tuberculosis, pancreatitis and heart failure. However, pleural effusion caused by multiple myeloma (MM) is extremely rare, with a frequency of only 6%, and is usually associated with benign conditions such as sepsis, pulmonary embolism, heart failure secondary to amyloidosis or chronic renal failure. Myelomatous pleural effusion (MPE) is rare, occurring in <1% of cases, and may result in a delayed diagnosis of MM (1). Although diagnostic procedures for MPE have not been well defined, the preferred methods defined in previously published studies are pleural fluid cytology or pleural biopsy via a thoracoscope, as well as bone marrow biopsy (2,3). Furthermore, diffuse osteoporosis or bone loss may be regarded as indirect diagnostic evidence for MPE. Despite considerable progress in the treatment of MM in the past decade, including a number of drugs with highly active agents, such as thalidomide, bortezomib and lenalidomide (4,5), the prognosis of MM patients with MPE remains poor and the median survival time is <4 months (6). The present study reports the case of a 78-year-old patient who initially presented with bilateral pleural effusion and elevated adenosine deaminase (ADA) activity, but was ultimately diagnosed with MPE.

## Case report

The study was conducted in accordance with the Declaration of Helsinki and with approval from the Ethics Committee of Taizhou People’s Hospital (Taizhou, China). Written informed consent was obtained from the patient. The 78-year-old patient was admitted to the Department of Respiratory Medicine at Taizhou People’s Hospital in March 2014, complaining of persistent dyspnea over the preceding 20 days. The patient had no history of smoking or lung disease. Following admission, a physical examination of the patient revealed a body weight of 62 kg, a height of 175 cm and a body temperature of 36.7°C. In addition, the patient had a pulse of 90 bpm, a respiratory rate of 22 bpm and a blood pressure of 120/80 mmHg. The patient appeared fatigued, however, no cyanosis of the lips was observed. A respiratory examination revealed decreased breath sounds in the bilateral lower hemithorax, with dullness of percussion.

**Figure 1:**
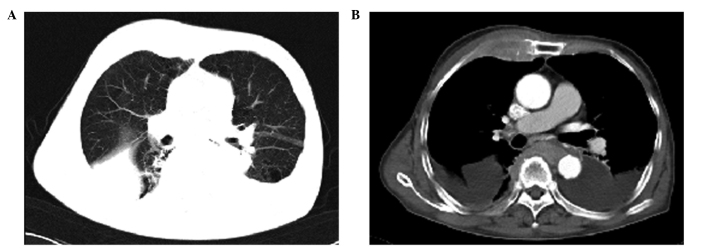
Computed tomography scans showing bilateral pleural effusion with no evident mass lesions in the (A) lungs and (B) mediastinal windows.

**Figure 2:**
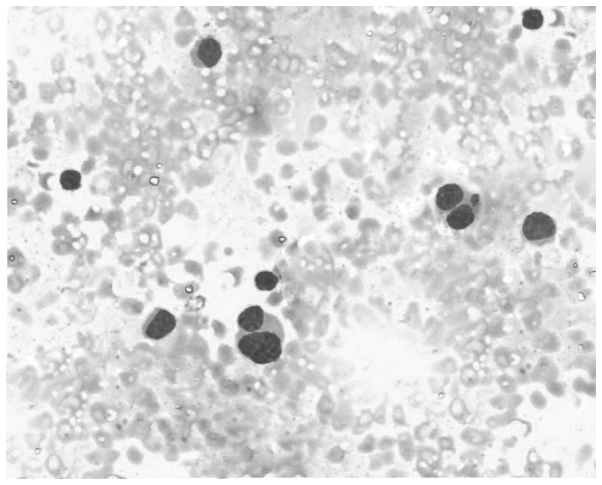
Cytological examination of the pleural fluid, showing atypical binucleate plasma cells (hematoxylin and eosin; magnification, ×400).

Laboratory results of the initial examination were as follows: Red blood cells, 4.59×10^12^/l (normal range, 4–5.5×10^12^/l); hemoglobin, 138 g/l (normal range, 120–160 g/l); white blood cells (WBC), 10.95×10^9^/l (normal range, 4–10×10^9^/l); platelets, 121×10^9^/l (normal range, 100–300×10^9^/l); and erythrocyte sedimentation rate, 12 mm/h (normal range, 0–15 mm/h). In addition, biochemical examinations revealed the following results: Total serum protein, 69.6 g/l (normal range, 66–87 g/l); albumin, 28.6 g/l (normal range, 35–54 g/l); globulin, 41 g/l (normal range, 20–40 g/l); IgD, 127 mg/l (normal range, 1–4 mg/l); IgG, 11.5 g/l (normal range, 8–17 g/l); IgA, 0.31 g/l (normal range, 0.7–4.0 g/l); IgM, 0.57 g/l (normal range, 0.4–2.3 g/l); C-reactive protein, 22.1 mg/l (normal range, 0–5.0 mg/l); serum carcinoembryonic antigen, 4.67 ng/ml (normal range, 0–6.5 ng/ml); neuron-specific enolase, 10.01 ng/ml (normal range, 0–20.0 ng/ml); and CYFRA 21-1, 2.19 ng/ml (normal range, 0.1–3.3 ng/ml). The levels of lactate dehydrogenase (LDH), electrolytes, glucose and fat, and the patient’s renal function were all normal and no Bence-Jones protein was detected in the patient’s urine. Computed tomography examination of the chest revealed bilateral pleural effusion and a small amount of pericardial effusion, but no evident mass lesion (Fig. 1A and B). Thoracentesis was performed and the pleural effusion was found to be exudative and slightly bloody. The pleural fluid was shown to contain 13.1×10^12^/l WBC (neutrophils, 41%; lymphocytes, 25%; mesothelial cells, 34%), 35.8 g/l total protein, 4,187 U/l LDH and an elevated total ADA level of 80 U/l (0–35 U/l). In addition, pleural fluid cytology revealed atypical plasma cells with binucleated forms (Fig. 2). The results of the mycobacterial and bacterial cultures were negative. The patient was ultimately diagnosed with MM (IgD type).

**Figure 3:**
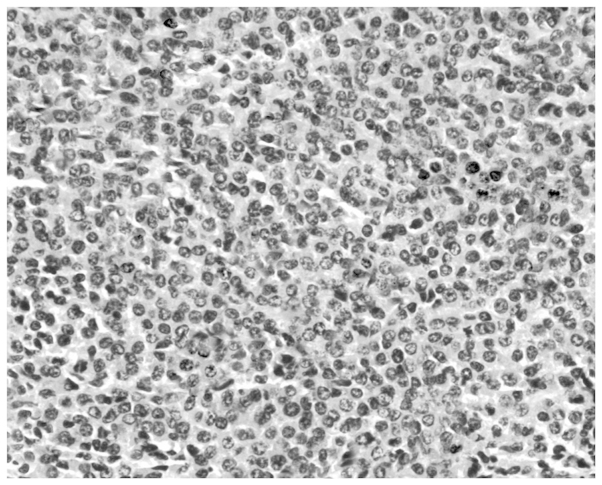
Bone marrow biopsy revealing active hyperplasia of the plasma and lymphoid cells (hematoxylin and eosin; magnification, ×400).

Following confirmation of the diagnosis, a bone marrow biopsy was performed, revealing active hyperplasia of the lymphoid plasma cells (Fig. 3). A plain radiograph showed degeneration in the thoracic and lumbar vertebra, and diffuse osteoporosis in the ilium, pubis and sciatic nerve. The patient was subsequently transferred to the Department of Hematology at Taizhou People’s Hospital to receive chemotherapy with CHOP (cyclophosphamide: 750 mg/m^2^, i.v., day 1; doxorubicin: 50 mg/m^2^, i.v., day 1; vincristine: 2 mg, i.v., day 1; and prednisolone: 100 mg, p.o., days 1–5) which was repeated every 21 days. Following two rounds of chemotherapy, the pleural effusion was reduced and partial remission of MM was achieved.

## Discussion

MM, also known as plasma cell myeloma, is a malignant plasma cell disorder that accounts for 1% of all malignant neoplasms and 10% of hematological malignant neoplasms (7). The main clinical pathological characteristics of MM are the clonal proliferation of plasma cells in the bone marrow and the overproduction of structurally homogeneous immunoglobulins. These changes eventually lead to a series of clinical symptoms, which include anemia, hemorrhage, recurrent infection, bone loss, hypercalcemia, renal failure, highly viscous hematic disease, nervous system damage and amyloidosis (8).

Fewer than 5% of MM patients present clinically with apparent extraosseous symptoms (9). Clinicopathological studies have revealed that the spleen, lymph nodes and liver are the most frequent sites of MM occurrence. However, the development of MM in the kidneys, central nervous system, skin, pleura, testes, pancreas, thyroid, adrenal glands and omentum have also been reported (10). Cases of MM in which the respiratory system is involved are extremely uncommon, particularly where bilateral pleural effusion with an elevated ADA activity is the only initial symptom. This rarity may result in a delayed diagnosis of MM. Pleural effusion occurs in ~6% of patients with MM during the course of the disease, while myelomatous pleural effusion (MPE) is even rarer, presenting in <1% of patients (11). Cho *et al* (2) retrospectively reviewed the records of 734 patients with MM and found that only 19 cases had been diagnosed with MPE. Kintzer *et al* (11) reported a study of 958 patients with MM in which the rate of accompanied pleural effusion was 6.1% (58/958), while only eight of these cases (0.8%) were diagnosed with MPE. A number of mechanisms have been proposed for the pathogenesis of pleural effusion in MM (12), which include the infiltration of the pleural fluid by malignant plasma cells or directly from adjacent tissues, nephrotic syndrome secondary to renal tubular infiltration with paraprotein, the development of glomerular damage and congestive heart failure secondary to amyloidosis, and lymphatic drainage obstruction by tumor infiltration.

Diagnosis of MM in the present study was based on the criteria of the International Myeloma Working Group (13). Increasing levels of awareness and improved diagnosis of MM have significantly increased the rate at which MPE has been reported (2,3,11,14). Although diagnostic procedures for MPE have not been well defined, the preferred methods in all published studies are pleural fluid cytology or pleural biopsy via a thoracoscope, as well as bone marrow biopsy (2,3). In addition, diffuse osteoporosis or bone loss may be regarded as indirect diagnostic evidence of MPE. To the best of our knowledge, the positive rate of pleural fluid cytology is low and closely associated with the competence of pathologists. Therefore, the diagnostic value of a pleural biopsy using a thoracoscope for MPE has been associated with a higher positive rate of diagnosis in previous studies (3,15).

In the present study, significantly elevated ADA activity was observed in the exudative pleural effusion of the patient. To the best of our knowledge, the majority of pulmonologists would interpret the present symptoms as tuberculous pleural effusion, even though no evidence of active pulmonary tuberculosis was found in the patient. However, elevated ADA activity in the pleural fluid has been associated with a number of malignant neoplasms, including non-Hodgkin’s lymphoma and breast cancer (14,17). Previous studies have also reported high levels of ADA in patients with MPE (2,3). Cho *et al* (2) hypothesized that a small fraction of myeloma cells express ADA on their surface, which may be associated with the high levels of ADA observed in MPE patients.

There have been major advances in the treatment of MM in the past decade, with a number of drugs emerging as highly active agents, including thalidomide, bortezomib and lenalidomide (4,5,18). However, the prognosis of MM patients with MPE remains poor and the median survival time is less than four months (6). Hematopoietic stem cell transplantation may improve response rates and prolong the median overall survival rate in MM patients; however, this treatment is not suitable for patients with MPE who are frequently characterized by an aggressive clinical course, as well as a bad performance status.

In conclusion, the occurrence of bilateral pleural effusion with an elevated ADA activity as the initial symptom in MM is extremely rare, and may result in a delayed diagnosis. In such cases, improved awareness of MM as a potential diagnosis and knowledge concerning the clinical presentation of MPE are key factors in ensuring timely diagnosis and effective intervention.
